# Immersive virtual reality for older adults with mild cognitive impairment, dementia, or cognitive frailty: a systematic review and narrative synthesis (2019–2025)

**DOI:** 10.1186/s12877-025-06957-8

**Published:** 2026-01-13

**Authors:** Kazumi Kubota, Tomohiro Katayama, Kei Takamaru, Yousuke Ishii, Leona Adachi, Ryunosuke Tanabe, Kosuke Tsubota

**Affiliations:** 1 Research Organization, Shimonoseki City University, 2‑1‑1, Daigaku‑cho, Shimonoseki, Yamaguchi, 751‑8510 Japan; 2 Department of Healthcare Information Management, The University of Tokyo Hospital, Tokyo, Japan; 3 Entertainment & Care Lab. Co, Ltd., Tokyo, Japan; 4 Graduate School, International University of Health and Welfare, Tokyo, Japan

**Keywords:** Virtual reality, Mild cognitive impairment, Dementia, Cognitive frailty, Older adults, Executive function, Dual‑task, Mobility, Anxiety, Systematic review

## Abstract

**Background:**

Immersive virtual reality (VR) is increasingly used to support cognition, mobility, and emotional well‑being in older adults with mild cognitive impairment (MCI), dementia, or frailty. Evidence is expanding but fragmented across small randomized and feasibility trials. We synthesized recent studies to clarify benefits, risks, and implementation considerations.

**Methods:**

Following PRISMA 2020, we searched PubMed and CINAHL from 1 January 2019 to 15 October 2025. Eligible studies enrolled adults aged ≥ 65 years with MCI, dementia, or frailty/cognitive frailty; delivered immersive or semi‑immersive VR via head‑mounted display or large‑screen projection (interactive tasks or 360° content); and reported cognitive, mobility, or emotional/behavioral outcomes in randomized, quasi‑experimental, or pre–post designs. Two reviewers independently screened and extracted data. Risk of bias was appraised with RoB 2 (randomized trials) or JBI tools (non‑randomized). Heterogeneity precluded meta‑analysis; we conducted a structured narrative synthesis.

**Results:**

Seventy records were identified (PubMed 28; CINAHL 42); after removing 9 duplicates, 61 records were screened, 24 full texts were assessed, and 13 studies were included (10 randomized; 3 feasibility/mixed‑methods). The most consistent improvements occurred in executive function and processing speed among participants with MCI or cognitive frailty; several trials also reported modest gains in global cognition. Multiple trials showed better Timed Up & Go and Berg Balance outcomes and enhanced anticipatory postural adjustments versus comparators. In residential care, immersive reminiscence and group VR reduced anxiety and apathy and were well tolerated. Adverse events were uncommon and mild; adherence was high with supervised delivery. Most randomized trials had some concerns for bias; one was at overall low risk.

**Conclusions:**

Immersive and semi-immersive VR interventions appear feasible for supervised delivery in older adults with MCI or cognitive frailty and may be associated with improvements in cognitive and mobility outcomes. Evidence for emotional and behavioral outcomes in institutional settings is promising but preliminary. Programs with adequate exposure (2–3 sessions/week for 8–12 weeks; ≥ 15 total hours), adaptive challenge, and supervision were most frequently associated with positive outcomes. Larger multicenter randomized trials with standardized outcomes and embedded implementation and economic evaluations are needed.

**Supplementary Information:**

The online version contains supplementary material available at 10.1186/s12877-025-06957-8.

## Background

Population aging is accelerating the need for engaging, scalable, and safe interventions that support both cognitive health and functional mobility in later life. Older adults with MCI, dementia, or frailty experience executive dysfunction, slowed processing, and impaired dual‑tasking that together reduce independence and elevate fall risk. Symptoms such as anxiety, apathy, and other behavioral changes further erode quality of life for individuals and caregivers and add to the burden on health and social care systems.

VR offers controlled, multisensory environments where cognitive tasks, motor practice, wayfinding, and personalised content can be combined and titrated. In recent years, immersive and semi‑immersive VR programs have been tested in outpatient rehabilitation, community settings, and residential aged care. Meta‑analyses suggest small‑to‑moderate gains in global cognition and attention and relatively larger effects on executive function when exposure is adequate [1–4]. VR may also enhance dual‑task performance, a determinant of falls in later life [5]. However, interventions vary widely in immersion level, content, dose, and delivery setting, and the evidence base is fragmented across small trials.

We therefore conducted a focused, up‑to‑date synthesis of immersive and semi‑immersive VR interventions for older adults with MCI, dementia, or cognitive frailty. Our objectives were to summarize effects on cognition, mobility, and emotional/behavioral outcomes; appraise risk of bias; and discuss practical considerations for nurse‑led, age‑friendly implementation. Because immersive VR programs in this field often integrate cognitive, motor, and psychosocial components within the same delivery platform and care settings, we structured the synthesis by outcome domain and population.

## Methods

### Protocol and reporting

This systematic review followed PRISMA 2020 guidance. A protocol was not registered. The review window was prespecified to capture contemporary VR technologies (1 January 2019 to 15 October 2025). The PRISMA 2020 checklist is provided as Additional files.

### Eligibility criteria

We included studies that:

- enrolled adults with mean or minimum age ≥ 65 years with mild cognitive impairment (MCI), dementia, or frailty/cognitive frailty;
- delivered immersive or semi‑immersive virtual reality (VR) via head‑mounted displays or large‑screen projection (interactive tasks or 360° immersive content such as reminiscence);
- reported outcomes in cognition (for example, Montreal Cognitive Assessment [MoCA], Mini‑Mental State Examination [MMSE], Trail Making Test, Digit Symbol, Stroop), mobility (for example, Timed Up & Go [TUG], Berg Balance Scale [BBS], gait speed, anticipatory postural adjustments), or emotional/behavioral health (for example, Geriatric Depression Scale, Hospital Anxiety and Depression Scale, anxiety or apathy measures);
- used randomized, quasi‑experimental, or pre–post designs.

We excluded augmented or mixed reality, non‑VR exergames, purely diagnostic or cross‑sectional studies, and populations outside scope (for example, acquired brain injury or Parkinson’s disease without cognitive impairment). We restricted inclusion to English‑language publications.

### Information sources and search strategy

We systematically searched PubMed and CINAHL for records published from 1 January 2019 to the last search date (15 October 2025). Search strategies were piloted to balance precision and recall and required “virtual reality” in the title to reduce off‑target retrieval. Filters for date and English language were applied when available and otherwise enforced during screening. The executable queries were:

- PubMed (searched 15 Oct 2025)

"virtual reality"[ti] AND (cognit*[tiab] OR memory[tiab] OR depression[tiab] OR anxiety[tiab] OR balance[tiab] OR gait[tiab]) AND (moca[tiab] OR mmse[tiab] OR "trail making"[tiab] OR "digit symbol"[tiab] OR stroop[tiab] OR "n-back"[tiab] OR gds[tiab] OR "phq-9"[tiab] OR hads[tiab] OR tug[tiab] OR "berg balance"[tiab] OR sppb[tiab] OR "chair stand"[tiab] OR "gait speed"[tiab]) AND (randomized[tiab] OR randomised[tiab] OR trial[tiab] OR pilot[tiab] OR feasibility[tiab]) AND ("2019/01/01"[dp]: "2025/12/31"[dp]).

AND ("mild cognitive impairment"[tiab] OR MCI[tiab] OR dementia[tiab] OR frail*[tiab]).

NOT (protocol[tiab] OR augmented[tiab] OR "mixed reality"[tiab] OR AR[tiab]).

- CINAHL (EBSCOhost; searched 15 Oct 2025)

TI ("virtual reality") AND AB (cognit* OR memory OR depression OR anxiety OR balance OR gait) AND AB (moca OR mmse OR "trail making" OR "digit symbol" OR stroop OR "n-back" OR gds OR "phq-9" OR hads OR tug OR "berg balance" OR sppb OR "chair stand" OR "gait speed") AND AB (randomized OR randomised OR trial OR pilot OR feasibility) AND AB ("mild cognitive impairment" OR MCI OR dementia OR frail*).

NOT AB (protocol OR augmented OR "mixed reality" OR AR) Limits applied: publication date 2019–2025; English (where available).

The searches returned 28 records from PubMed and 42 from CINAHL. All results were exported on the search date and merged into a single Microsoft Excel file. De‑duplication was conducted in Excel: potential duplicates were first flagged automatically based on exact matches in key bibliographic fields (e.g., title, first author, year, and journal, and DOI/PMID when available) and were then manually verified by two reviewers to confirm true duplicates and address minor citation discrepancies. Any uncertainties were resolved by consensus. The de‑duplicated records were then entered into the screening workflow.

### Selection process

**Fig. 1:**
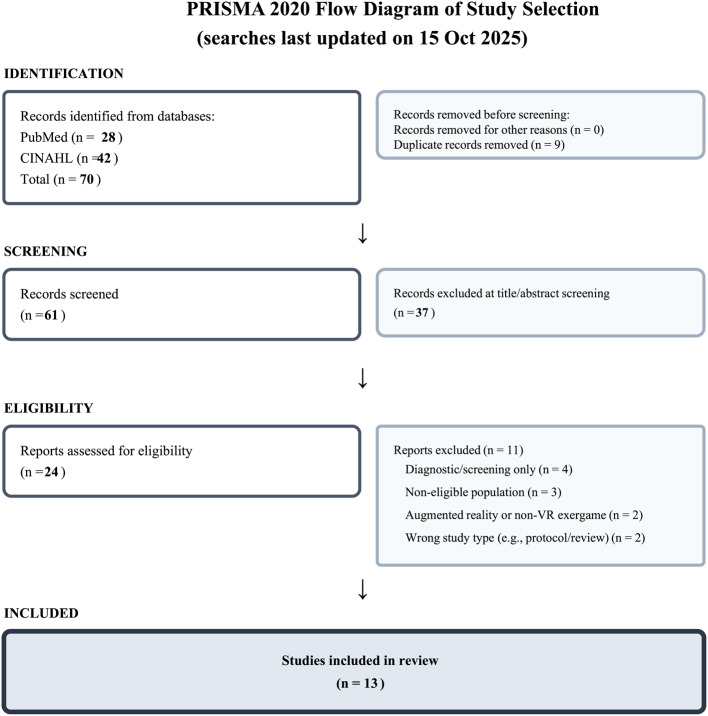
PRISMA 2020 flow diagram of study selection. Database searches (PubMed and CINAHL) were last updated on 15 Oct 2025. Of 70 records identified, 9 duplicates were removed and 61 records were screened. 24 full‑text reports were assessed for eligibility, 11 were excluded with reasons, and 13 studies were included in the review

Two reviewers independently screened titles/abstracts and then full texts in duplicate; disagreements were resolved by consensus. Interrater agreement was monitored; a formal kappa was not calculated. The PRISMA 2020 flow diagram is shown in Fig. 1.

### Data collection and data items

Two reviewers independently extracted design and setting, sample characteristics, VR modality and device, dose (session length, frequency, duration), comparator, outcomes and timepoints, adherence, adverse events, and main findings using a piloted form.

### Risk of bias assessment

Randomized trials were appraised using the Cochrane Risk of Bias 2 tool (domains D1–D5). Non‑randomized or single‑group studies were appraised using Joanna Briggs Institute (JBI) critical appraisal tools. Overall judgement followed the highest domain risk across applicable domains. Study‑level assessments are summarized in Table 2.

### Synthesis methods

Given heterogeneity in interventions, comparators, and outcome measures, we did not perform a meta‑analysis. A structured narrative synthesis was conducted, organised by domain (cognition, mobility, emotional/behavioral) within populations (MCI, dementia, cognitive frailty). Direction of effects was summarized using vote counting based on statistical significance and consistency across outcomes/timepoints. Reported effect sizes were described narratively where available.

## Results

### Study selection

The searches identified 70 records. After removing 9 duplicates, 61 records were screened at title/abstract; 24 full‑text reports were assessed for eligibility; 11 were excluded with reasons; and 13 studies were included (Fig. 1).

### Study characteristics (Table 1)

Of the 13 included studies, 10 were randomized trials and three were feasibility or mixed‑methods studies. Studies were conducted across Asia, Europe, and Australia in outpatient rehabilitation, community programs, and residential aged care. Most interventions used fully immersive head‑mounted displays; several used semi‑immersive large displays for group sessions. Typical dosing was 2–3 sessions per week, 20–45 min per session, for 4–12 weeks. Cognitive outcomes included MoCA/MMSE, Trail Making Test A/B, Digit Symbol, and Stroop; mobility measures included TUG, BBS, gait speed, and anticipatory postural adjustments; emotional/behavioral outcomes included anxiety, apathy, and behavioral symptoms. Full characteristics are provided in Table 1.

**Table 1:**
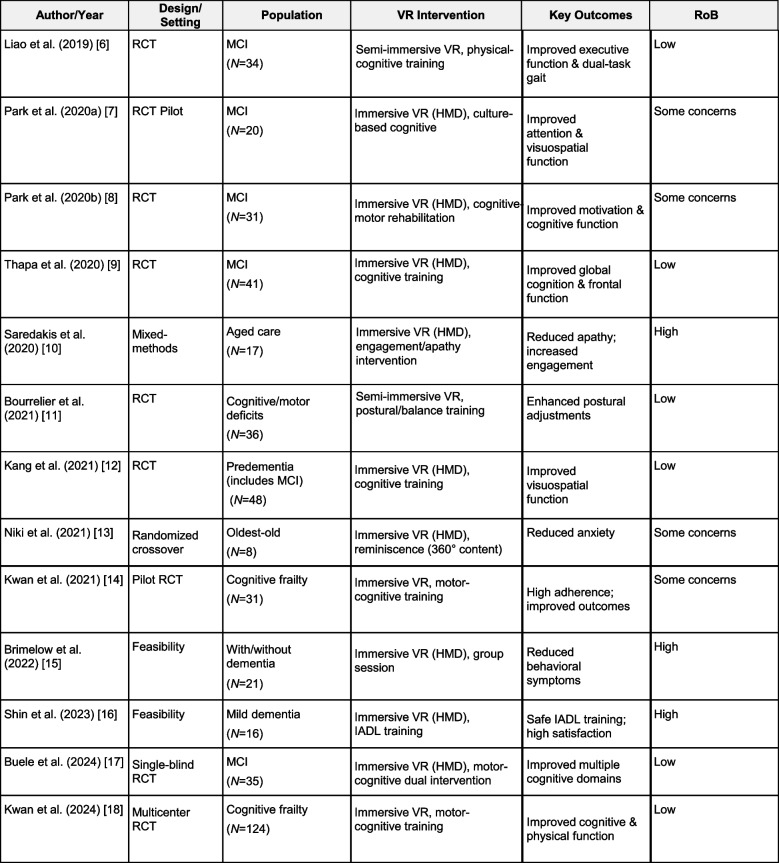
Characteristics of included studies (*n*=13) [6–18]

*RCT* Randomized Controlled Trial, *MCI *Mild Cognitive Impairment, *HMD* Head-Mounted Display, *RoB *Risk of Bias, *IADL *Instrumental Activities of Daily Living. RoB indicates overall risk-of-bias judgment (RoB 2 for randomized trials; JBI appraisal for non-randomized/feasibility designs).

### Risk of bias (Table 2)

Most randomized trials had some concerns, driven by allocation concealment, lack of participant blinding, and outcome measurement; one trial was at overall low risk. Non‑randomized studies were at higher risk due to design limitations. Detailed assessments are shown in Table 2.

**Table 2:**
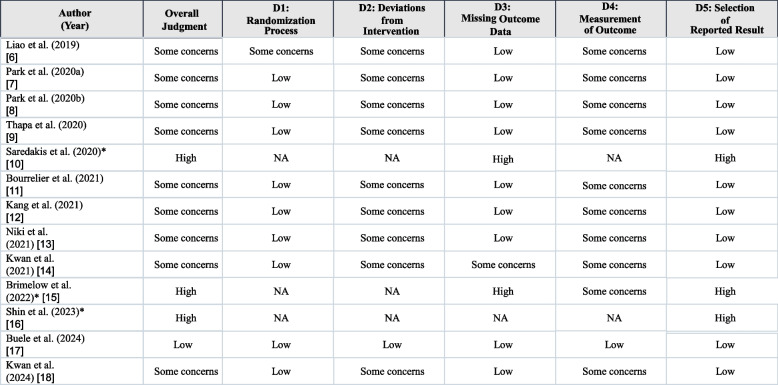
Risk-of-bias summary by study [6–18]

Randomized trials were appraised using RoB 2 (domains D1–D5). Non-randomized/feasibility studies were appraised using JBI critical appraisal tools; for these studies, RoB 2 domains are marked as NA and only the overall judgment is reported. Overall judgment reflects the highest risk across applicable domains

Cognitive outcomes Across randomized trials in MCI or cognitive frailty, immersive or semi‑immersive VR generally improved executive function and processing speed relative to controls. VR‑augmented physical and cognitive training improved dual‑task executive performance and global cognition in MCI [6, 9]. A randomized controlled study reported enhanced motivation with cognitive gains using VR‑based cognitive‑motor rehabilitation [7], and a randomized pilot trial demonstrated feasibility and better attention/visuospatial performance with culture‑based immersive content [8]. A single‑blind randomized trial combining motor training with VR‑based cognitive tasks showed advantages across multiple domains [17]. In a multicenter randomized trial of cognitive frailty, motor‑cognitive VR training improved cognitive measures compared with usual activities [18]; a pilot randomized trial in community‑dwelling participants supported feasibility and suggested benefit [14]. In predementia, fully immersive training improved visuospatial function with concurrent changes in functional connectivity [12]. These findings align with meta‑analytic evidence of small‑to‑moderate improvements in global cognition and attention and relatively larger effects on executive function with adequate exposure [1–4].

### Mobility outcomes

Mobility benefits were also observed. In randomized trials involving older adults with cognitive and motor deficits, VR training enhanced anticipatory postural adjustments versus comparison activities [11]. In MCI, programs integrating VR with physical training reduced TUG times and improved BBS relative to controls [6, 9]. In cognitive frailty, motor‑cognitive VR training improved functional mobility in a multicenter randomized trial [18]. These results accord with evidence that VR can improve dual‑task performance in older adults [5].

### Emotional and behavioral outcomes

In residential aged care, immersive reminiscence reduced anxiety in a randomized crossover study of the oldest‑old and was well tolerated without serious adverse effects [13]. Group‑based immersive sessions were feasible and associated with reductions in behavioral and psychological symptoms over multiple sessions [15]. A mixed‑methods study in nursing homes reported improved apathy and engagement following immersive VR [10]. Early feasibility work in mild dementia demonstrated the safety and acceptability of instrumental activities of daily living training using fully immersive VR [16]. Overall, emotional outcomes are promising but based on small samples and, in some cases, non‑randomized designs.

### Adherence, acceptability, and safety

Adherence was generally high in supervised programs, aided by structured onboarding and group formats. Adverse events were uncommon and mild—mainly transient nausea, dizziness, or eye strain. Semi‑immersive large‑screen delivery tended to reduce cybersickness while maintaining engagement among frailer residents.

### Synthesis summary (Table 3)

The strongest and most consistent benefits were seen in executive function and dual‑task mobility in MCI or cognitive frailty, with modest gains in global cognition in several trials. In residential care, immersive reminiscence and group VR showed promising reductions in anxiety and apathy with good acceptability. A domain‑by‑population overview is provided in Table 3.

**Table 3:**
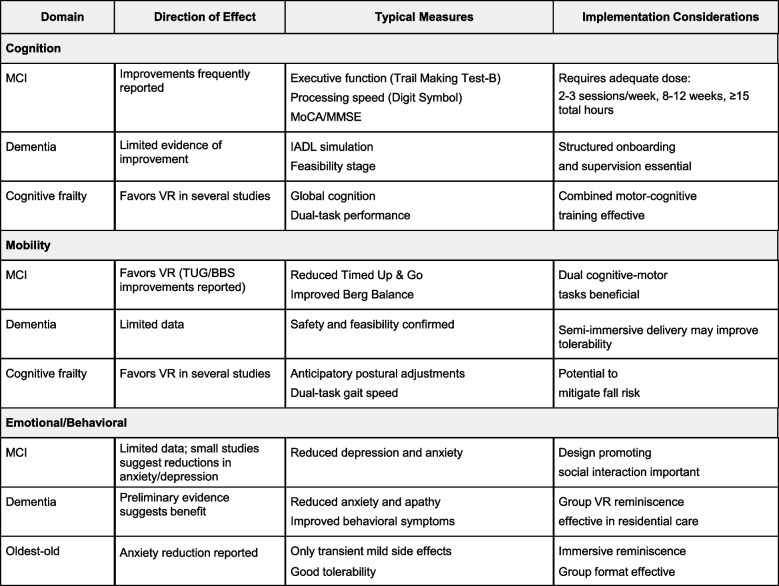
Domain-by-population effect overview

Direction of effect was summarized using vote counting based on statistical significance at reported timepoints. This approach summarizes direction but not magnitude of effects

## Discussion

This review suggests that immersive and semi‑immersive VR can be useful adjuncts for older adults living with MCI, dementia, or cognitive frailty. Improvements in executive function and processing speed are clinically meaningful because they underpin planning, attentional control, and safe ambulation. Gains in TUG, BBS, and anticipatory postural adjustments indicate potential to mitigate fall risk, complementing evidence that VR can enhance dual‑task performance [5–7, 9, 11, 18]. In institutional settings, immersive reminiscence and other engaging VR content appear acceptable and can reduce anxiety and apathy, aligning with non‑pharmacological approaches to behavioral and psychological symptoms of dementia [10, 13, 15].

Across domains, benefits were more apparent when programs provided adequate exposure (2–3 sessions weekly over 8–12 weeks, accumulating ≥ 15 total hours), adaptive challenge, and supervision, consistent with prior meta‑analyses [1–4]. Implementation in age‑friendly systems can begin with nurse‑led group programs featuring structured onboarding, safety screening (vestibular sensitivity, fall risk, visual/hearing needs), and infection control for shared headsets. Semi‑immersive large‑screen delivery can broaden reach for participants who do not tolerate head‑mounted displays.

Future research should prioritize larger and longer multicenter randomized trials with active comparators, standardized outcome batteries (for example, MoCA/MMSE, Trail Making Test, Digit Symbol, TUG, BBS), adequate dose (for example, ≥ 24 sessions or ≥ 15 total hours), and 6–12‑month follow‑up. Implementation science frameworks and economic evaluations in community and long‑term care should be embedded to inform scale‑up and sustainability.

### Limitations

This review has several limitations. First, we searched two databases (PubMed and CINAHL) and restricted inclusion to English‑language publications. Relevant studies indexed in other databases or published in other languages may have been missed, introducing selection and language bias. We also required “virtual reality” to appear in the article title to improve precision; this decision likely reduced sensitivity and may have excluded eligible trials that described VR only in the abstract or methods. Grey literature and trial registries were not searched, so publication and time‑lag bias cannot be ruled out.

Second, interventions, comparators, and outcomes were heterogeneous. Programs varied by immersion level (head‑mounted versus semi‑immersive), content (cognitive tasks, motor‑cognitive training, reminiscence), dose, and supervision. Many trials combined VR with concurrent physical or cognitive training, making it difficult to isolate the specific contribution of VR. Outcome measures and timepoints were inconsistently reported, and follow‑up was generally short. Because of this heterogeneity we did not pool effects; our vote‑counting approach summarizes direction but not magnitude and is sensitive to small‑study findings.

Third, the overall certainty is limited by study quality and size. Most randomized trials had “some concerns” for risk of bias (for example, unclear allocation concealment, lack of participant blinding), and the non‑randomized studies were at higher risk due to design limitations. Samples were small and often single‑centre, limiting precision and generalisability—particularly to people with more advanced dementia, to community settings without supervision, and to health systems outside the study regions. Adherence and adverse events were variably reported, so tolerability may be under‑ or over‑estimated.

Fourth, although our original eligibility emphasised interactive VR, we included some studies using 360° immersive reminiscence. We clarified this decision a priori during screening, but it introduces conceptual heterogeneity across interventions. In addition, hardware and software generations differed across studies; findings may not directly translate to newer devices.

Finally, the review protocol was not registered, and we did not conduct a formal GRADE assessment of evidence certainty or explore small‑study/publication bias quantitatively. These factors should be considered when interpreting the conclusions and underscore the need for larger, well‑reported, and longer‑term randomized trials with standardized outcomes and follow‑up.

## Conclusions

Immersive and semi-immersive VR interventions appear feasible for supervised delivery in older adults with MCI or cognitive frailty and may be associated with improvements in executive function and dual-task mobility outcomes. Evidence for reductions in anxiety and apathy in residential care is promising but remains preliminary, given small sample sizes, heterogeneity, and risk-of-bias concerns in many included studies. Larger, longer multicenter randomized trials with standardized outcomes, adequate dose, longer follow-up, and embedded implementation and economic evaluations are needed before broad scale-up.

## Supplementary Information

Supplementary Material 1

Supplementary Material 2

Supplementary Material 3

Supplementary Material 4

## Data Availability

All data supporting the findings of this study are contained within the article. The PRISMA 2020 checklist and two CSV files (exported database results) are provided as supplementary materials. Additional materials (e.g., search logs, full-text exclusion lists with reasons, and risk-of-bias matrices) are available from the corresponding author upon reasonable request.

## Abbreviations

BBS: Berg Balance Scale
HADS: Hospital Anxiety and Depression Scale
HMD: Head-Mounted Display
IADL: Instrumental Activities of Daily Living
JBI: Joanna Briggs Institute
MCI: Mild Cognitive Impairment
MMSE: Mini-Mental State Examination
MoCA: Montreal Cognitive Assessment
PRISMA: Preferred Reporting Items for Systematic reviews and Meta-Analyses
RCT: Randomized Controlled Trial
RoB 2: Cochrane Risk of Bias 2 tool
TMT: Trail Making Test
TUG: Timed Up & Go
VR: Virtual Reality
