# Supplementary material for: Immersive virtual reality for older adults with mild cognitive impairment, dementia, or cognitive frailty: a systematic review and narrative synthesis (2019–2025)
Source: BMC Geriatr. 2026 Jan 13;26:189. doi: 10.1186/s12877-025-06957-8 (PMC12888722; doi:10.1186/s12877-025-06957-8)
Supplement: Supplementary file 1 — Supplementary Material 1 [file 12877_2025_6957_MOESM1_ESM.pdf]

Supplementary file 1. Full search strategies

Manuscript: *Immersive virtual reality for older adults with mild cognitive impairment, dementia, or cognitive frailty: a systematic review and narrative synthesis (2019–2025)*

Database: PubMed
Platform: NCBI PubMed
Date searched: 15 October 2025
Time period limited to: 1 January 2019–31 December 2025 (via date range in query)
Language: English (filter applied when available; otherwise enforced during screening)
Search query (as executed):

"virtual reality"[ti]
AND (cognit*[tiab] OR memory[tiab] OR depression[tiab] OR anxiety[tiab] OR balance[tiab] OR gait[tiab])
AND (moca[tiab] OR mmse[tiab] OR "trail making"[tiab] OR "digit symbol"[tiab] OR stroop[tiab]
OR "n-back"[tiab] OR gds[tiab] OR "phq-9"[tiab] OR hads[tiab] OR tug[tiab]
OR "berg balance"[tiab] OR sppb[tiab] OR "chair stand"[tiab] OR "gait speed"[tiab])
AND (randomized[tiab] OR randomised[tiab] OR trial[tiab] OR pilot[tiab] OR feasibility[tiab])
AND ("2019/01/01"[dp] : "2025/12/31"[dp])
AND ("mild cognitive impairment"[tiab] OR MCI[tiab] OR dementia[tiab] OR frail*[tiab])
NOT (protocol[tiab] OR augmented[tiab] OR "mixed reality"[tiab] OR AR[tiab])

Database: CINAHL
Platform: EBSCOhost
Date searched: 15 October 2025
Limits applied in EBSCOhost: Publication date 2019–2025; English (where available)
Search query (as executed):
TI ("virtual reality")
AND AB (cognit* OR memory OR depression OR anxiety OR balance OR gait)
AND AB (moca OR mmse OR "trail making" OR "digit symbol" OR stroop OR "n-back"
OR gds OR "phq-9" OR hads OR tug OR "berg balance" OR sppb OR "chair stand"
OR "gait speed")
AND AB (randomized OR randomised OR trial OR pilot OR feasibility)
AND AB ("mild cognitive impairment" OR MCI OR dementia OR frail*)
NOT AB (protocol OR augmented OR "mixed reality" OR AR)

Notes

- The phrase "virtual reality" was required in the **title** (PubMed: [ti]; CINAHL: TI) to increase precision and reduce retrieval of non-VR digital interventions.
- Records were screened in English; English-language filters/limits were applied when available and otherwise enforced during screening.
